# Morphological Biomarkers in the Amygdala and Hippocampus of Children and Adults at High Familial Risk for Depression

**DOI:** 10.3390/diagnostics12051218

**Published:** 2022-05-12

**Authors:** Bradley S. Peterson, Tejal Kaur, Maria Andrea Baez, Ronald C. Whiteman, Siddhant Sawardekar, Juan Sanchez-Peña, Xuejun Hao, Kristin W. Klahr, Ardesheer Talati, Priya Wickramaratne, Myrna M. Weissman, Ravi Bansal

**Affiliations:** 1 Children’s Hospital Los Angeles, Keck School of Medicine of the University of Southern California, Los Angeles, CA 90024, USA; ssawardekar@chla.usc.edu (S.S.); rabansal@chla.usc.edu (R.B.); 2 Division of Child and Adolescent Psychiatry, Columbia College of Physicians and Surgeons, New York, NY 10032, USA; tejalkaurmd@gmail.com (T.K.); mbaez@montefiore.org (M.A.B.); 3 Center for Developmental Neuropsychiatry, New York State Psychiatric Institute, New York, NY 10032, USA; rwhiteman@gradcenter.cuny.edu (R.C.W.); jp.sanchezpena@gmail.com (J.S.-P.); xuejun.hao@nyspi.columbia.edu (X.H.); kwklahr@gmail.com (K.W.K.); 4 Department of Psychology, The Graduate Center at the City University of New York, New York, NY 10032, USA; 5 Department of Psychiatry, Columbia College of Physicians and Surgeons, New York, NY 10032, USA; at2071@cumc.columbia.edu (A.T.); pjw1@columbia.edu (P.W.); weissman@nyspi.columbia.edu (M.M.W.); 6 The Mailman School of Public Health, New York, NY 10032, USA

**Keywords:** biomarker, endophenotype, surface morphology, familial risk, depression, hippocampus, amygdala, magnetic resonance imaging

## Abstract

Major Depressive Disorder (MDD) is highly familial, and the hippocampus and amygdala are important in the pathophysiology of MDD. Whether morphological markers of risk for familial depression are present in the hippocampus or amygdala is unknown. We imaged the brains of 148 individuals, aged 6 to 54 years, who were members of a three-generation family cohort study and who were at either high or low familial risk for MDD. We compared surface morphological features of the hippocampus and amygdala across risk groups and assessed their associations with depression severity. High- compared with low-risk individuals had inward deformations of the head of both hippocampi and the medial surface of the left amygdala. The hippocampus findings persisted in analyses that included only those participants who had never had MDD, suggesting that these are true endophenotypic biomarkers for familial MDD. Posterior extension of the inward deformations was associated with more severe depressive symptoms, suggesting that a greater spatial extent of this biomarker may contribute to the transition from risk to the overt expression of symptoms. Significant associations of these biomarkers with corresponding biomarkers for cortical thickness suggest that these markers are components of a distributed cortico-limbic network of familial vulnerability to MDD.

## 1. Introduction

Anatomical MRI studies have reported cortical thinning [1,2] and abnormal volumes in the hippocampus and amygdala [3,4,5,6,7,8,9,10,11,12,13,14,15] in persons suffering from major depressive disorder (MDD). Postmortem studies of individuals with MDD have reported smaller neuronal soma in the hippocampus [16]; fewer glial cells in the amygdala [17]; reduced density of astrocytes in the hippocampus [18]; and reduced N-acetylaspartate, an index of neuronal integrity, in both the hippocampus [19,20] and amygdala [19]. Diffusion tensor imaging studies have demonstrated abnormalities in white matter tracts connecting cortical and limbic structures [21,22], suggesting that anatomical and cellular disturbances in the amygdala and hippocampus may represent abnormalities within wider corticolimbic circuits. 

Most of these studies have reported abnormalities in brain structure and function in already-affected individuals. Studies of already-affected persons, however, are unable to distinguish whether abnormalities represent vulnerability to illness or a consequence of having illness, including the effects of chronic stress and treatment [23]. Therefore, we undertook an imaging study of individuals in a 3-generation cohort who were at either high or low familial risk for developing MDD, with the aim of discerning whether abnormalities in the hippocampus and amygdala represent either a vulnerability to developing MDD or the consequences of prior illness. The use of a multigenerational family design has the substantial advantage of being able to identify true endophenotypes for MDD that are heritable, state-independent, and transmitted through unaffected family members, and that co-segregate with the illness in families [24,25]. These attributes confer a greater likelihood of associating with the genetic determinants of familial MDD, which currently are thought to comprise numerous predisposing common genetic variants; each of these confers a small overall contribution to risk, requiring multiple risk genes to act in concert to manifest as overt illness [26].

We previously reported, in the same participants, a putative endophenotype for familial MDD that consisted of prominent thinning of the entire lateral surface of the right hemisphere and mesial wall of the left [1]. Cortical thickness correlated inversely with current symptoms of depression and inattention. The validity of the marker status of cortical thinning was supported by its presence in the high-risk participants who had never developed MDD. We hypothesized, in the present study, that we would detect morphological abnormalities in the hippocampus and amygdala in individuals at elevated familial risk for MDD, and these abnormalities would be present in high-risk individuals without prior depression, thereby representing a true vulnerability marker for depressive illness. We also explored whether those abnormalities were associated with the previously identified thinner cortices in these participants, which would suggest that they represent a corticolimbic circuit of risk for MDD.

## 2. Materials and Methods

Participants in this study included individuals from a 3-generation, prospectively ascertained cohort who were followed in 5 waves of clinical data collection, in which the fifth wave, presented here, also included MRI scanning. The full description of study design, sample selection, and assessments of the three generations has been reported previously [27]. Members of Generation 1 (G1) consisted of persons with either MDD or no discernible lifetime history of depression. Those with MDD were selected from an outpatient clinic for the pharmacologic treatment of depression, and they had moderate to severe MDD with impairment in functioning. Nondepressed controls were selected from the same community as the patients and were required to have no lifetime history of psychiatric illness, based on several interviews. After Wave 2, the spouses of 2 participants in the G1 control group developed a first major depression. These 2 spouses and their 4 offspring were reassigned to the depressed group in G1. We did not remove or reassign group membership for any participants in G2 or G3 if they developed any clinical disorders, because their diagnoses were the study outcomes.

The fifth wave of assessments was performed in a partial sample of the second generation (G2) and third generation (G3) who agreed to MRI scanning. We defined participants at ‘‘high-risk’’ for developing MDD to be members of Generation 2 (G2) and Generation 3 (G3) who were biological descendants of the MDD group in G1, and those at ‘‘low-risk’’ as the G2 and G3 biological descendants of the unaffected control group in G1. We defined “lifetime MDD” status as a binary variable where participants had either never had an episode of MDD or had one or more episodes, as ascertained by assessment throughout the 5 waves. Risk status using our definition did not change when a G2 or G3 member developed a depressive episode.

All assessment and imaging procedures were undertaken with approval of the Columbia University/New York State Psychiatric Institute Institutional Review Board. Written consent was obtained from all participants over 18 years old; written parental consent and written youth assent was obtained for all participants 17 years or younger. Inclusion criteria for each of the 5 waves of assessment were an age of 6 years or over, and the absence of current psychotic symptoms that would interfere with the consent process. Exclusion criteria for Wave 5 assessments were current pregnancy or ferromagnetic implants. We scanned 148 G2 and G3 descendants from 56 distinct G1 families.

### 2.1. Participant Characterization

Full methodological details for the assessments performed at Wave 1 (baseline), Wave 2 (year 2), Wave 3 (year 10), and Wave 4 (year 20) follow-ups have been described previously [27,28]. Assessment procedures were kept similar across the waves, with few exceptions, to avoid introducing bias from method variation. G1 participants and their spouses, offspring, and grandchildren (if applicable) were interviewed independently, with interviewers blind to the clinical status of participants in the previous generations.

The diagnostic interviews across all waves were conducted using a semi-structured diagnostic instrument (the Schedule for Affective Disorders and Schizophrenia–Lifetime Version for adults [27], and a child version of the instrument for youth [29]. Diagnostic interviews were performed by doctoral and master’s level interviewers and best-estimate diagnosis was determined using an independent review by a child psychiatrist and child psychologist who were blind to the participant’s risk group. Agreement between evaluators was good to excellent as determined by interrater Cohen’s kappa coefficients (two clinicians) at Wave 4 of 178 randomly selected participants from all generations: MDD, 0.82; dysthymia, 0.89; anxiety disorder, 0.65. Diagnostic interviews in the fifth wave were generally performed on the same day as the MRI, and always occurred within 1 month of the MRI scan.

Assessments in the fifth wave of assessments, at the time of MRI scanning, also included: the Children’s Depression Rating Scale-Revised [30] or the Hamilton Depression Rating Scale [31] to measure depressive symptoms in children or adults, respectively; and the Revised Children’s Manifest Anxiety Scale [32] or Hamilton Anxiety Rating Scale [33] to measure anxiety symptoms in children and adults, respectively. An index of the severity of depressive or anxiety symptoms across children and adults was computed by calculating a z-score for each participant, and then combining those z-scores across age groups into a single variable for each symptom domain, i.e., “z-depression” or “z-anxiety.” The z-scores were, thus, normalized according to age group.

We also used the Global Assessment Scale (GAS) (54) to estimate the degree of functional impairment for each participant. It was completed at all waves by the persons making the best-estimate diagnoses. The GAS is rated on a scale ranging from 0 to 100 and estimates a person’s current overall functional adjustment based on all available information. A child version of the GAS (C-GAS) [34] was used for participants of 6–17 years of age. Lower scores on the GAS indicate greater overall functional impairment.

### 2.2. MRI Scanning

High-resolution MRI scans were obtained using a Siemens Sonata 1.5 Tesla scanner (Siemens AG, Munich, Germany). Head positioning was standardized using canthomeatal landmarks. Brain scans were acquired using a 3D MP-RAGE sequence (repetition time, 24 ms; echo time, 2.96 ms; 45° flip angle; 256 × 192 matrix; field of view, 30 cm; 2 excitations, slice thickness 1.2 mm; 124 contiguous slices encoded for sagittal slice reconstruction with voxel dimensions of 1.17 × 1.17 × 1.2 mm).

During scan acquisition, we closely monitored data from each sequence in real time as images were reconstructed and displayed on the scanner console. Any visible motion triggered a repeat of the sequence. Image quality within individual pulse sequences was assessed in more detail during preprocessing within 48 h of the scan, as described below. Every effort was made to bring back for repeat scanning, with the relevant pulse sequences, any participants who had images containing a motion artifact. Additional quality control for anatomical images included ratings of image sharpness, ringing, contrast-to-noise ratio of subcortical nuclei, and gray/white matter interface.

### 2.3. Image Processing

We corrected large-scale variations in image intensity using a validated algorithm developed at the Montreal Neurological Institute [35]. We removed extracerebral tissues using an automated tool for extracting the brain [36]. Connecting dura was then removed manually on each slice in the sagittal view and checked in the orthogonal views. The brainstem was transected at the pontomedullary junction.

All brain regions were traced manually on Sun Ultra 10 workstations using ANALYZE 8.0 software (Biomedical Imaging Resource, Mayo Foundation), together with software developed in-house, while blind to participant characteristics and hemisphere (images were randomly flipped in the transverse plane before preprocessing and then reversed prior to statistical analysis).

We measured whole brain volume for use as a covariate to control for global scaling effects in statistical analyses of overall regional volumes. This measure included not only gray and white matter, but also cerebrospinal fluid within the ventricles and cortical sulci, to ensure the exclusion of any possible confound of age-related effects of tissue atrophy with this general measure of body scaling [37]. Detailed procedures for defining cortical thickness measures are provided in a prior publication [1].

Methods for defining the hippocampus and the amygdala followed previously published algorithms [38]. The rostral extent of the amygdala coincided with the most anterior section in which the anterior commissure crossed the midline. We determined the transition between the amygdala and hippocampus with a line connecting the inferior horn of the lateral ventricle with the amygdaloid sulcus or, when the sulcus was not obvious, with a straight horizontal line connecting the inferior horn of the lateral ventricle with the surface on the uncus [39]. The most posterior section was the last section in which the crus of the fornix and the fimbria of the hippocampal formation could be delineated. Region definitions for all participants were reviewed for accuracy by a PhD candidate in cognitive neuroscience (RCW) trained by an expert in neuroanatomy (BSP).

### 2.4. Deformation-Based Surface Analyses

Our previously validated procedures for surface analysis [40] were customized to accommodate independent analysis of the right and left structure for each hippocampus and amygdala. Briefly, a rigid-body similarity transformation with global scaling was used to register the entire brain of each participant with the template brain, thereby inherently controlling for whole brain volume in all subsequent measurements. Each region (right and left hippocampus and amygdala) was then coregistered to the corresponding template region using a rigid body transformation. This second transformation created a refined registration by which to compare surfaces of isolated regions. Each region was next warped to the corresponding region of the template using a high-dimensional, nonrigid warping algorithm based on the principles of fluid-flow dynamics [41]. Warping permitted point-to-point matching of the surfaces of the regions for each participant with the surfaces of the corresponding regions in the template brain. The high-dimensionally warped images were then unwarped to the refined rigid body registration while maintaining the point-to-point correspondences established by the non-linear warping. These point correspondences permitted calculation of the signed Euclidean distance of each surface point from the corresponding point on the surface of the template region. Euclidean distances were compared statistically across risk groups to identify areas of protrusion or indention, from which we inferred underlying greater or lesser volumes, respectively, along the surface of each amygdala and hippocampus.

Boundaries for various subfields of the hippocampus and nuclei of the amygdala of the template brain were estimated using a modified version of a digital brain atlas [42] with a widely used parcellation scheme and nomenclature [43]. The atlas was registered to the 4 template structures (right and left hippocampus and amygdala) using a 3-dimensional nonlinear transformation based on voxel intensity. After smoothing this image manually, a simplified outline of the hippocampal subfields and amygdala nuclei, respectively, were overlaid on the respective template structures, to aid localization of findings for individual subfields of the hippocampus or nuclei of the amygdala.

#### 2.4.1. The Template Brain

We used a single representative brain as a template rather than an averaged brain from many participants, because a single brain has sharp CSF-to-gray matter and gray matter-to-white matter tissue interfaces, thereby improving the accuracy of registration [40,44,45]. Averaging images for a template blurs these boundaries and increases registration errors that are important when distinguishing subtle effects across populations. In addition, precise surface morphometry requires a brain with smooth gray and white matter surfaces that are devoid of topological defects, which cannot be reconstructed by averaging brains from many individuals. Finally, the amygdala and hippocampal surfaces have considerable variability between persons, even after whole brain coregistration, and require further registration, which can be accomplished with precision only when using a single reference region as a template.

To select the most appropriate, representative template brain for surface morphometry, we first selected as a preliminary template of the brain of a low-risk participant whose age and overall brain size were nearest the group average. The brains for all remaining participants in the sample were coregistered to that preliminary template, the point correspondences across their surfaces were determined as detailed above, and the distances of those points from the corresponding points on the preliminary template surface were calculated. The brain for which all points across its surface were closest (in the least squares sense) to the average of the distances across those points for the entire sample was selected as the final template that was morphologically most representative of all brains in the cohort. We also note that our findings are robust with respect to the specific template used, as we have shown previously that findings generated when using randomly selected brains from our sample as the template differ minimally from those when using the most representative brain as the template [46].

#### 2.4.2. Index for Right-Hemisphere Cortical Thinning

We created an index for the degree of right-hemisphere cortical thinning, previously reported in this cohort, by averaging cortical thickness over voxels of the right hemisphere where the effect of risk group was statistically significant. We used this index to assess whether cortical thickness was associated with surface measures of the hippocampus and amygdala. 

### 2.5. Statistical Analyses

#### 2.5.1. Conventional Volumes

We used repeated measures analysis in the context of a mixed effects model [47], employing an unstructured covariance matrix between the left and right hemispheres for each region and adjusting for hemisphere, to test the overall associations of risk group with volume and to assess the interaction of risk with either region or hemisphere. Covariates included sex, age, and a quadratic term (age^2^) to capture curvilinear associations with age. We did not include age- or sex-by-risk-group interactions, as these interactions were not significant.

#### 2.5.2. Surface Morphometry

We compared high- and low-risk groups on the signed Euclidean distances at each point on the surfaces of the amygdala and hippocampus while covarying for age, age^2^, and sex. We also assessed risk-group effects while excluding all participants who had a lifetime history of MDD, to determine which of the risk effects was fully independent of prior MDD. We also excluded all participants taking psychotropic medications at the time of scan to ensure that risk effects were independent of medication effects. We also assessed whether our findings were stable when redefining the categorical “risk status” variable as either (a) one or both participant’s parents had a lifetime history of depression, or (2) no parents had a lifetime MDD history.

We also assessed the stability of our findings by assessing risk-group effects for a representative voxel in the dorsal head of the left hippocampus while controlling for age, age^2^, sex, and familial relatedness across participants. We used two independent methods to control for relatedness—one that employed Generalized Estimating Equations (GEE) assuming an exchangeable correlation structure across participants within the same family [48], and another that employed a linear mixed-effects model and incorporated a random effect for family [47].

We further assessed whether age, sex, and history of lifetime MDD modified the effect of risk group on surface morphological measures by testing, at each voxel, the interaction of these variables in statistical models; these models included the variables age, age^2^, sex, the interaction term, and each of the interaction’s two component main effects. In both groups combined, we also assessed the associations of surface measures with symptom severity, defined as the sum of z-scores for clinician ratings of depression and anxiety in each participant, while covarying for age, age^2^, sex, and risk group.

Finally, we assessed the association of our index of right-hemisphere cortical thinning with surface measures in each region while covarying for age, age^2^, and sex. We also included this index as a covariate in our base model for risk-group effects, to determine which biomarkers for risk in the hippocampus and amygdala were most associated with right-hemisphere thinning.

#### 2.5.3. Correction for Multiple Comparisons

We corrected *p*-values in each statistical map using a topological False Discovery Rate (FDR) threshold of *p* < 0.05 and color-coded corrected probability values at each point across the surface of the reference regions.

## 3. Results

The frequencies of lifetime MDD and anxiety were significantly greater in the high-risk than low-risk group, though the severity ratings of current depression and anxiety symptoms were similar across groups (Table 1).

**Figure 1:**
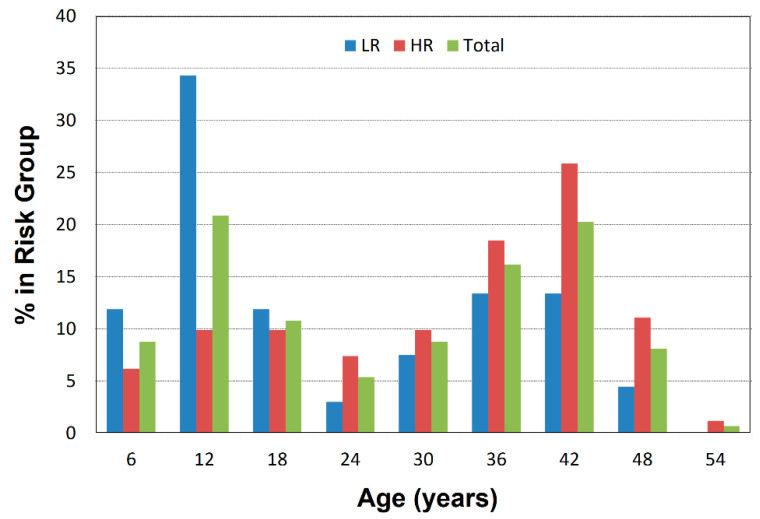
Age Distributions for High- and Low-Risk Groups. The percentage of participants in each risk group who are found in each age bin is plotted against age in 6-year bins. Plots are shown separately for the low-risk group, high-risk group, and the total sample. The 7–12-year age bin has more low-risk than high-risk children.

The distributions of ages in each risk group were similar, except for a larger proportion of school-aged children in the low-risk group (Figure 1).

### 3.1. Risk Effects

Risk group was not associated with overall volumes of the whole brain, hippocampus, or amygdala (Table 2). We detected no significant interaction of risk status with hemisphere on regional volumes (risk-by-hemisphere F = 0.003, *p* = 0.95 (amygdala); F = 0.17, *p* = 0.68 (hippocampus)).

**Figure 2:**
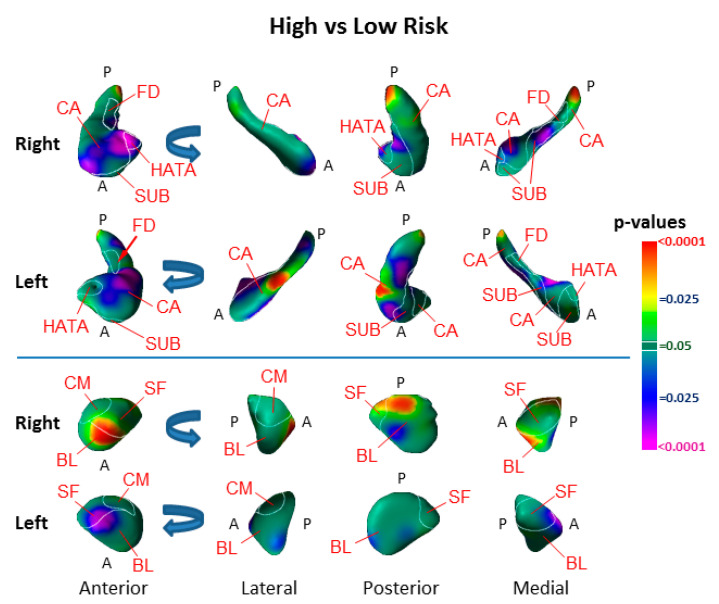
Main Effect of Risk Group on Surface Morphologic Features. The right and left hippocampus and amygdala are shown in their anterior, lateral, posterior, and medial views, with the overlaid cytoarchitectonic map shown in the thin white outlines. Anterior (A) and posterior (P) positions are indicated. Arrows in the rotational views show the direction of rotation. The statistical significance (probability values) of differences in surface morphology across risk groups (81 high-risk, 67 low-risk participants) are color coded at each point on the hippocampal and amygdala surfaces. The color bar indicates the color coding for *p*-values associated with the main effect of risk group, with warmer colors (yellow and red) indicating protruding surfaces, presumably from larger underlying volumes, and cooler colors (blue and purple) indicating indented surfaces and presumably smaller underlying volumes in those regions. *p*-values are thresholded at *p* < 0.05 after correction for multiple comparisons using FDR. The statistical model included the main effect of risk group and the covariates of age, age^2^, and sex. Abbreviations: A—anterior; P—posterior; CA—cornu ammonis; SUB—subiculum; FD—fascia dentata; HATA—hippocampal–amygdaloid transition area; BL—basolateral nucleus; SF—superficial nucleus; CM—centromedial group.

Surface analyses detected the effect of risk group as inward deformations in the head of both hippocampi and the anterior surface of the left amygdala, and protrusions of the lateral body of the left hippocampus and the anterior and dorsal posterior aspect of the right amygdala (Figure 2).

**Figure 3:**
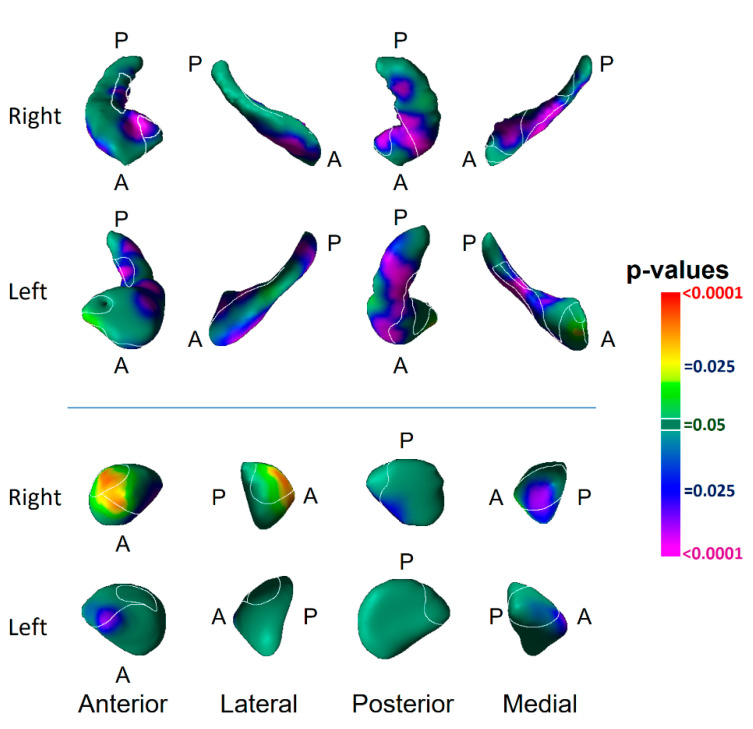
Maps of the Main Effect of Parental Depression on Surface Morphological Features of the Hippocampus and Amygdala. Groups were redefined as either having at least one parent with a lifetime history of depression (N = 94) or having no parent with a lifetime history (N = 54). Using this alternative definition, we reassigned group status for 19 of the 67 LR participants and 6 of the 81 HR participants. Findings were similar to those comparing high- vs. low-risk (Figure 2).

**Figure 4:**
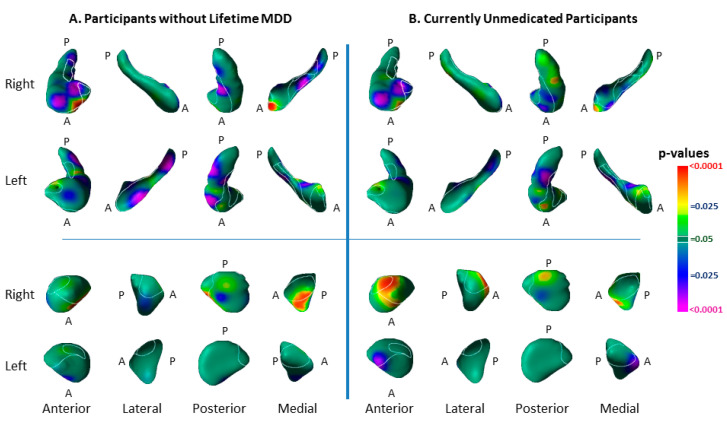
Risk-group Effect Distinct from the Effect of Lifetime Major Depressive Disorder and Medication Use. Panel (**A**): The risk-group effect (32 HR vs. 55 LR) is shown when excluding all participants who had a lifetime history of MDD. Findings for the hippocampus are very similar to the effects when including all participants (Figure 1 of the main text), demonstrating that the surface morphological features for that structure are genuine risk effects, as they are detected in offspring of persons with MDD but who, themselves, have never had depressive illness. Many of the findings in the amygdala shown in Figure 1 of the main text, however, are not statistically significant in this subgroup analysis, either because those effects derive from the effects of lifetime MDD or because of the reduced statistical power in this analysis because of the smaller numbers of participants in this subgroup analysis. Panel (**B**): The risk-group effect (57 HR vs. 59 LR) is shown when excluding all participants who were taking psychotropic medications at the time of MRI scanning. The effect of risk group is similar to the effects detected when including all participants (Figure 1). These findings demonstrate that the risk effects reported Figure 1 are independent of medication effects. The statistical models included the main effect of risk group, and the covariates of age, age^2^, and sex. *p*-values are thresholded at *p* = 0.05 after FDR correction for multiple comparisons.

The findings did not differ substantially when redefining the risk group as having a lifetime history of depression in one or both participants’ biological parents (Figure 3) or when controlling for familial relatedness using either GEE or linear mixed effects (Table 3). The differences between risk groups in hippocampal surface morphology generally persisted in analyses that excluded participants with a lifetime history of MDD; most group differences in the amygdala, however, did not persist (Figure 4). The findings were little changed when excluding participants taking psychotropic medications (Figure 4, Table 4).

### 3.2. Modifier Effects

**Figure 5:**
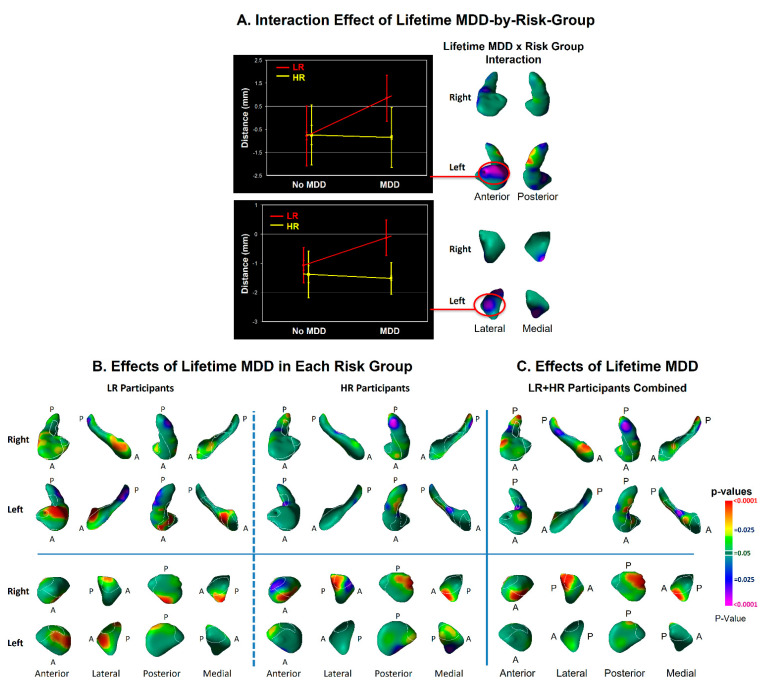
Effects of Lifetime Depressive Illness on Surface Morphologic Features. Panel (**A**): The effect of the lifetime MDD-by-risk-group interaction on surface morphological features is shown in the lateral and medial views of each hippocampus and amygdala. The interaction is plotted for representative points of the left hippocampus and left amygdala where this interaction is statistically significant, showing that the interaction derives from relative protrusions in the surface of low-risk (LR) participants at those locations. Panel (**B**): The effects of lifetime MDD are shown for high- and low-risk groups separately to help further understand the significant interaction of risk group with lifetime illness shown in Panel (**A**). The statistical model included the main effect of lifetime MDD and the covariates of age, age^2^, and sex. Lifetime illness was associated in both risk groups (HR N = 81, LR N = 67) with inward deformations in the tail and body of the hippocampus, but the low-risk participants also had associations with prominent protrusions in the head of the left hippocampus and lateral surface of the left amygdala. Panel (**C**): The main effect of lifetime MDD is shown for both LR and HR groups combined. The statistical model included lifetime MDD, risk group, age, age^2^, and sex. lifetime MDD was associated in both risk groups with protrusions in the dorsolateral head and indentations of the tail of both hippocampi, and with protrusions in posterolateral and anteromedial surface of the right amygdala. Most of the protrusions of the right amygdala associated with lifetime MDD were also present in similar locations for the effect of risk group (Figure 2).

We detected significant interactions of risk group with lifetime MDD in the dorsal head of the left hippocampus and lateral surface of the left amygdala (Figure 5) that derived from protrusions at those locations in low-risk participants with a lifetime history of MDD (Figure 5).

**Figure 6:**
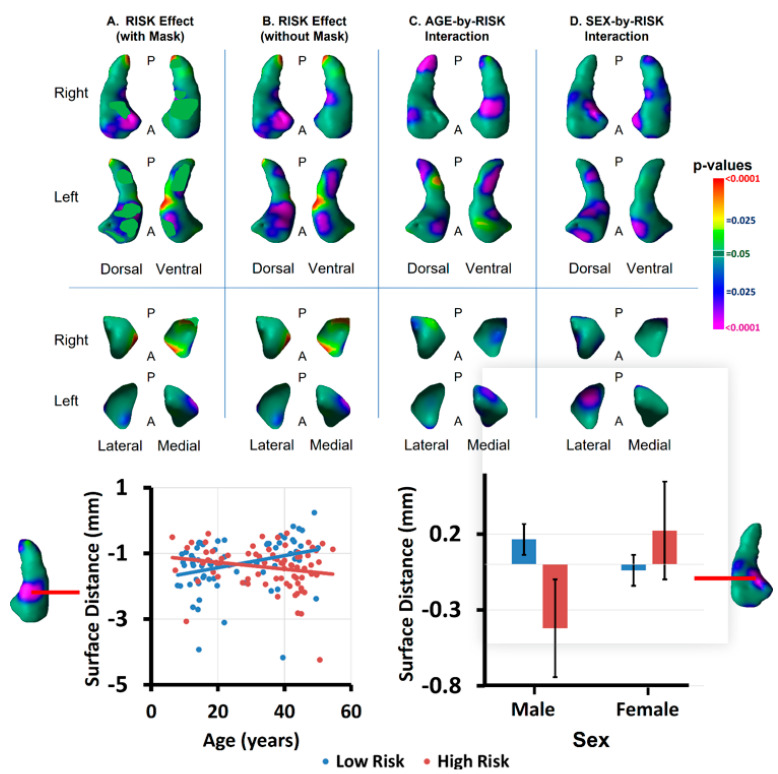
Interactions of Risk Group with Age and Sex, and Residual Risk Effects. Panel (**A**): The effect of risk group is shown only in locations without significant age- or sex-by-risk-group interactions, which have been masked out of the statistical map. The significant main effects of risk group that remain are, therefore, independent of age and sex and are valid for all ages and both sexes. The independent variables were age, age^2^, sex, and risk group. Panel (**B**): The effect of risk group is shown without masking any interaction effects (as shown in Figure 2). The independent variables were age, age^2^, sex, and risk group. Panel (**C**): The effect of the age-by-risk-group interaction is shown at each voxel. Significant voxels were used to generate a mask that was then applied to the statistical map of Panel (**A**). Independent variables were age, age^2^, sex, risk group, and age-by-risk-group. The scatterplot at the bottom left indicates that the interaction derives from increasing protrusion with age in the LR group but greater indentations with age in the HR group. Panel (**D**): The effect of the sex-by-risk-group interaction is shown at each voxel. Significant voxels were used to generate a mask that was then applied to the statistical map of Panel (**A**). Independent variables were age, age^2^, sex, risk group, and sex-by-risk-group. The bar graph on the bottom right indicates that the interaction derives from greater indentations in the HR males than in the other participants.

Age significantly moderated the effect of risk group (as assessed by the age-by-risk-group interaction) in the anterior body and tail of both hippocampi and the posterior portions of both amygdalae (Figure 6). The interaction of age with risk group at those locations derived from increasing protrusion with age in the low-risk group and increasing indentation with age in the high-risk group (Figure 6).

Sex significantly moderated the effect of risk in portions of the head body of both hippocampi and lateral surface of the left amygdala (Figure 6). The interaction of sex with risk group in those locations derived from greater indentations in males of the high-risk group than in other participants (Figure 6). When masking out these regions of significant interaction for age and sex, the main effect of risk group remained in the anterior head of both hippocampi and the anterior and medial surfaces of the left amygdala (Figure 6).

### 3.3. Associations with Symptom Severity

**Figure 7:**
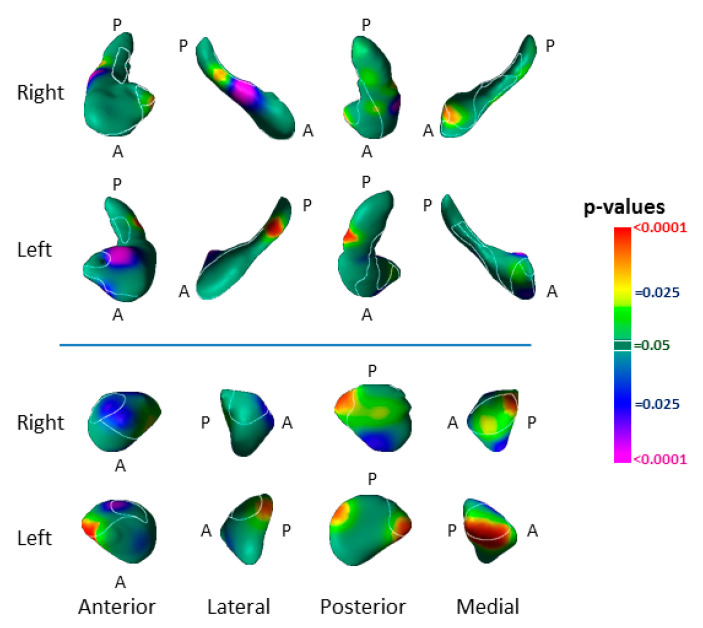
Associations with Current Symptom Severity. Shown here are the associations of symptom severity with surface morphology of the hippocampus and amygdala in both groups combined (HR N = 78, LR N = 67). We defined current symptom severity as the sum of z-scores for depression and anxiety measures in each participant. Independent variables included z-score for symptom severity, age, age^2^, sex, and risk group. Greater symptom severity accompanied inward deformation of the head and body and protrusion of the tail and the hippocampus, as well as inward deformation of the anterior surface of the left amygdala and protrusion the medial surface of both amygdalae.

In analyses of both groups combined, we detected significant associations of surface measures with symptom severity. More severe symptoms accompanied inward deformation of the dorsal head and anterior body of the left hippocampus and lateral body of the right hippocampus, as well as with inward deformation of the anterior surface of the right amygdala and protrusion of the medial aspect of the left (Figure 7).

### 3.4. Associations with Right-Hemisphere Cortical Thinning

**Figure 8:**
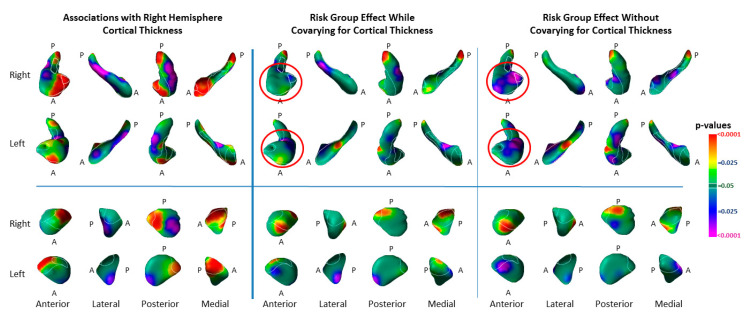
Associations of Right-Hemisphere Cortical Thickness with Surface Morphologic Features of the Hippocampus and Amygdala. Left Panel: The statistical significance (*p*-values) for the associations of an index for right-hemisphere cortical thickness with surface measures on each structure are shown. Significant positive associations are in warm colors. Inverse associations are in cool colors. The index for right-hemisphere cortical thickness was calculated by averaging cortical thickness measures at each voxel within the voxels of the lateral surface of the right hemisphere, where thickness differed significantly between the high and low-risk groups. Statistical models included the independent variables cortical thickness, risk group, age, age^2^, and sex. Associations are shown for high- and low-risk groups combined (HR (64) and LR (65)). Middle Panel: This shows the effects of risk group while covarying for the average cortical thickness in voxels of the cortex where thickness differed significantly between risk groups, as well as covarying for age, age^2^, and sex. Right Panel: For ease of comparison with the middle panel, this shows the same effects without covarying for cortical thickness (also shown in Figure 1). The magnitude of cortical thinning accounted for most of the variance of the risk-group effect for indentations in the hippocampus (red circles), but not in the amygdala. Number of participants: covarying for cortical thickness—HR 63, LR 65; without covarying for cortical thickness—HR 81, LR 67.

The index of cortical thinning accounted for the majority of variance in risk-group effects within the hippocampus, but not in the amygdala (Figure 8).

### 3.5. Medication Effects

**Figure 9:**
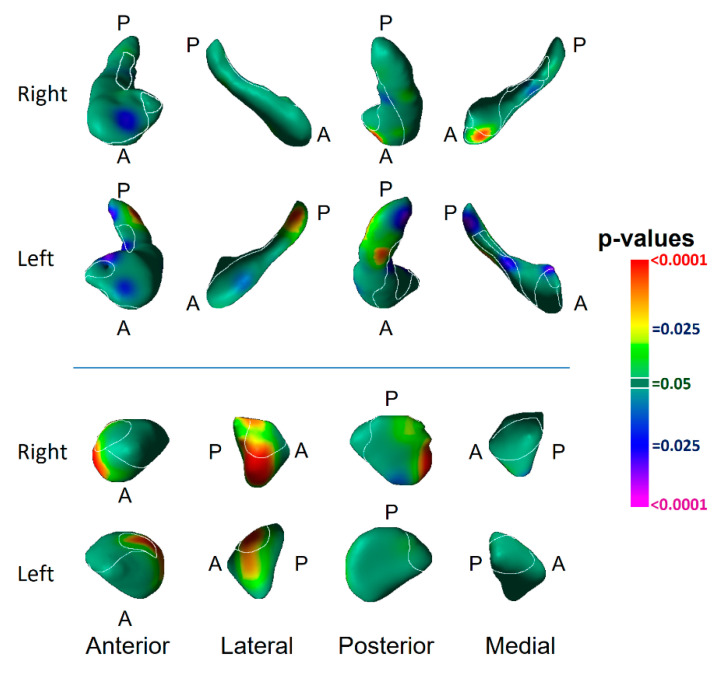
The Effects of Medication Use. Medication use at the time of MRI scanning was associated predominantly with inward deformations of the head, body, and tail of the hippocampus and of the lateral amygdala. None of the locations where we detected significant effects of medication were in locations where we detected significant effects of risk group or lifetime MDD, suggesting that our findings for risk group and lifetime MDD are likely independent of the effects of medication. We included only HR participants (N = 77) in this analysis, as only 6 LR participants were currently taking psychotropic medication. Covariates included age, age^2^, and sex. Findings were identical when including Lifetime MDD as a covariate. Of the 77 HR participants, 46 had lifetime MDD, 32 had no history of MDD, 20 were currently taking psychotropic medication, and 57 were not taking medication at the time of MRI scanning.

The effects of psychotropic medication use (Table 4), while covarying for lifetime MDD, included slight inward deformations of the dorsal head, medial body and tail of both hippocampi, and protrusions of the of the lateral surface of both amygdalae (Figure 9). None were in locations where we detected risk-group effects (Figure 2).

## 4. Discussion

We identified vulnerability biomarkers for MDD involving inward deformations of the head of both hippocampi and the medial surface of the left amygdala, as well as protrusion of the lateral body of the left hippocampus and the anterior and dorsal posterior aspect of the right amygdala (Figure 2). The inward deformations of the head of the hippocampus were present in those who never had MDD (Figure 4), indicating they are state-independent and suggesting they are strong candidates for an MDD endophenotype [24,25]. 

We also detected associations with lifetime MDD in both high- and low-risk participants (Figure 5). MDD-associated features common to both risk groups included inward deformations of the hippocampal tail and protrusions of the lateral and inferior medial aspect of the right amygdala. The amygdala findings were in locations where we detected the vulnerability marker for MDD, but at a magnitude above and beyond the effects of risk group, as lifetime MDD effects even persisted when controlling statistically for the effects of risk group (Figure 5). The similar location but statistical independence of risk and illness effects suggests that protrusion of the right amygdala constitutes a morphological vulnerability for MDD, but even greater protrusion may transform the vulnerability into overt illness. Alternatively, the greater protrusions in the amygdala associated with lifetime illness could be a consequence of MDD that exaggerates the endophenotypic marker for MDD risk. Prospective longitudinal imaging studies are needed to determine which of these two potential explanations is correct.

A greater severity of depression and anxiety symptoms accompanied more prominent inward deformations in the dorsal head and anterior body of the left hippocampus, as well as in the lateral body of the right hippocampus (Figure 7), in locations near the inward deformations identified as an effect of risk group (Figure 2). These findings suggest that a greater spatial extent of the vulnerability marker in the hippocampal head into the anterior body of the hippocampus could contribute to the development of anxiety and depression symptoms. More severe symptoms also accompanied inward deformation of the anterior surface of the right amygdala and protrusion of the medial aspect of the left (Figure 7)—in the same locations as the findings for risk-group effects in the amygdala, but opposite in direction. These opposing directions of effects suggest that the risk-group effects in the amygdala could contribute to resiliency in those at elevated familial risk for MDD [49], and the extent to which those risk-group effects are absent determines to what extent symptoms manifest. We note that lifetime MDD in the LR group was also significantly associated with enlargement of the head of the left hippocampus and of the anterior surface of the left amygdala (Figure 5), findings that differed significantly from those in the HR group (Figure 5). Because protrusion of the hippocampal head was associated with lower symptom severity, this protrusion in the LR participants with prior MDD could represent an enduring neuroplastic compensatory response to prior illness.

We assessed whether the markers we detected in the hippocampus and amygdala are likely components of a larger corticolimbic circuit of vulnerability for MDD. Greater right-hemisphere cortical thickness was significantly associated with proportionally greater protrusion of the anterior and medial head of the hippocampus, indentation of the lateral surface of the hippocampus, and bilateral protrusion of the medial surface of the amygdala (Figure 8). These findings indicate that right-hemisphere thinning was associated with bilateral indentations of the hippocampal head, both of which—cortical thinning and indentations of the hippocampal head—we have identified as putative biomarkers for risk vulnerability in familial MDD. Controlling for right-hemisphere thickness in our analyses of risk-group effect eliminated the hippocampal vulnerability marker, demonstrating the presence of shared variance in the right-hemisphere cortical thickness measures and surface measures in the head of the hippocampus. Taken together, hese findings strongly suggest the presence of a cortical–hippocampal circuit endophenotype of risk for developing familial MDD.

Age significantly moderated the effect of risk group (as assessed by the age-by-risk-group interaction) in the anterior body and tail of both hippocampi and the posterior portions of both amygdalae, deriving from increasing protrusion with age in the low-risk group and increasing indentation with age in the high-risk group (Figure 6). This modifying effect was adjacent to the locations of risk effect in both the hippocampus and amygdala, suggesting that the increasing indentation with age may represent a spatial extension of the risk markers into adjacent tissue as participants age. Longitudinal studies are needed to confirm this interpretation of the interaction, however. 

Sex significantly moderated the effect of risk in portions of the head and body of both hippocampi and the lateral surface of the left amygdala. The significant sex-by-risk-group interaction in these locations derived from greater indentations in males of the high-risk group relative to other participants (Figure 6). The greater indentation at these locations in males represents an exaggeration of the morphological biomarker for risk for MDD that we identified in our overall sample; moreover, it may suggest why these males are at elevated familial risk for illness, when males, in general, are relatively protected from developing MDD, having a relative risk of MDD that is approximately half that of females [50].

### 4.1. Hippocampus Subfields in the Pathogenesis of Depression

Both the vulnerability endophenotype and the marker for prior depressive illness consisted of inward deformations within the cornu ammonis (CA) subfield of the hippocampus (Figure 2). The vulnerability marker was most prominent in the CA head of the hippocampus, whereas the markers for prior illness were located primarily in the hippocampus CA body and tail. The CA, subiculum, and fascia dentata (FD) span the longitudinal axis of the hippocampus, and each participates in the processing of a distinct component of memory. The subiculum supports memory retrieval, the FD and CA3 classify those memories into patterns (“pattern separation”), the CA3 completes the recognition of those patterns in relation to stored memories (“pattern completion”), and the CA1 integrates these inputs within the hippocampus [51]. The specific function of each subregion further relates to its location along the longitudinal axis of the hippocampus. The anterior portions of these subfields project prominently to medial prefrontal and orbitofrontal cortices, as well as to the amygdala, to mediate the emotional content of cognitive processes [52]. The posterior portion of the hippocampus projects to the anterior cingulate, posterior cingulate, and retrosplenial cortices which, together, primarily mediate the non-emotional components of memory [52,53]. The risk endophenotypes we detected most prominently and definitively in the anterior CA and head of the hippocampus were significantly associated with right-hemisphere cortical thickness to constitute a cortical–hippocampal circuit endophenotype of risk for familial MDD; this suggests that this circuit may contribute to deficits in the processing of the emotional content of cognitive processes and memories that could both predispose patients to, and perpetuate, depressive illness.

### 4.2. Amygdala Nuclei in the Pathogenesis of Depression

Familial risk for MDD was associated with inward deformation of the anterior border of the superficial (SF) and basolateral (BL) nuclei of the left amygdala and protrusion in these same nuclei of the right amygdala (Figure 2). Bilateral protrusions in the BL nucleus were further associated with more severe symptoms (Figure 7), and in the right BL with lifetime MDD in the HR group (Figure 5). The SF nucleus selectively extracts the social from non-social value of incoming sensory cues, particularly in the processing of facial expressions [54]; meanwhile, the BL reciprocally projects to widespread cortical and subcortical regions that receive the sensory input of social cues, and to striatal regions that govern behaviors guided by these sensory stimuli [55,56,57]. Together, the BL and SF nuclei subserve the comprehension of social context and the modulation of affective states with goal-directed actions, impairments which could produce social and functional deficits that predispose patients to, and perpetuate, depression. The BL of the amygdala and head of the hippocampus, moreover, project reciprocally to one another and jointly modulate emotional processes [52,58]. Vulnerability markers for MDD were present in both these regions and provide further support for our contention that abnormalities within a distributed corticolimbic circuit are responsible for functional deficits that predispose patients to familial MDD.

### 4.3. Relationship to Prior Studies

Previously published studies have reported either reduced or normal overall volumes of the hippocampus in already-affected individuals with MDD compared to healthy controls [4,10,11]. Studies of the amygdala have reported either volume enlargement [6,12] or reduction [3,7,14]. Overall volumes, however, are insensitive to the detection of abnormalities in anatomical subregions, whereas studies that measure local surface features are more sensitive in detecting effects in subregions [59]. The overall volumes in our sample were similar across risk groups, despite the presence of significant group differences in subregions. Indeed, all prior published studies of MDD that we could identify that employed surface measures have detected inward deformations in subregions of these structures, including the anterior hippocampus and subiculum [13,60,61,62,63] and basolateral amygdala [63]; this is consistent with our findings for risk biomarkers in each structure. Prior studies of unaffected individuals at familial risk for depression [64,65,66] have reported overall volume reductions in the hippocampus. Our study extends these prior findings by identifying the regional specificity of volume reductions in the hippocampus and by showing that these findings are trait disturbances in those at high familial risk, and are likely components of a larger cortical–hippocampal circuit of risk for familial MDD. 

## 5. Limitations and Conclusions

Ascertainment bias in generation 1 could account for some of our findings in generations 2 and 3 and limit their generalizability, because the G1 group was not derived from representative samples of individuals with, or without, MDD. Despite this limitation, our findings suggest that inward deformations (local volume reductions) in the dorsal anterior head of the hippocampus are a component of a cortical–hippocampal circuit endophenotype of risk for familial MDD, consistent with the known functions and anatomical connectivity of these hippocampal subregions. Morphological abnormalities in the amygdala were not sufficiently independent of prior MDD to designate them as endophenotypes for risk. Posterior extension of the inward deformations into the body of the hippocampus seem to contribute to the presence of overt symptoms of depression. Our findings also suggest that risk-group effects in the amygdala could contribute to resiliency in persons who are at elevated familial risk for MDD. Only longitudinal follow-up assessments, however, can conclusively demonstrate that the risk effects we identified using MRI confer greater or lesser risks for developing MDD than does family history alone, and that the illness effects we identified are a consequence of new-onset illness. Those follow-up assessments are underway.

## Figures and Tables

**Table 1:** Demographic and Clinical Characteristics of Study Participants.

	Total Sample			Children			Adults		
Characteristic	High-Risk	Low-Risk	*p*-Value	High-Risk	Low-Risk	*p*-Value	High-Risk	Low-Risk	*p*-Value
Age	34.3 (12.7)	25.2 (13.3)	**0.0001**	13.1 (3.69)	13.3 (2.76)	0.788	38.4 (9.23)	35.5 (9.72)	0.127
Sex	35M:46F	31M:36F	0.742	8M:5F	16M:15F	0.395	27M:41F	15M:21F	0.505
GAS/CGAS	78.0 (9.48)	80.9 (9.85)	0.082	78.7 (9.689)	79.9 (7.621)	0.701	77.7 (9.80)	82.7 (9.73)	**0.015**
SES	35.2 (7.38)	34.0 (6.24)	0.301	32.9 (5.520)	34.0 (5.592)	0.534	35.7 (7.66)	34.0 (6.83)	0.281
PPVT IQ	101.6 (14.54)	102.7 (11.72)	0.689	109.9 (9.45)	101.9 (12.45)	0.103	100.0 (14.91)	103.5 (11.12)	0.296
Z-score Anxiety Severity	0.086 (0.903)	−1.094 (0.138)	0.275	0.370 (0.976)	−0.146 (0.988)	0.149	0.036 (0.889)	−0.066 (1.188)	0.634
Z-score Depression Severity	0.063 (1.115)	−0.072 (0.843)	0.425	−0.021 (1.40)	0.007 (0.836)	0.936	0.079 (1.07)	−0.143 (0.855)	0.299
Lifetime MDD (yes, no)	46.32	12.55	**0.0001**	1.11	0. 31	0.279	45.21	12.24	**0.001**
Lifetime Anxiety (yes, no)	42.36	19.48	**0.002**	4.8	5.26	0.201	38.28	14.22	0.056
Current MDD (yes, no)	2.76	0.67	0.189	0.12	0. 31	n/a	2.64	0.36	0.416
Current Anxiety (yes, no)	6.72	4.63	0.472	1.11	1.30	0.485	5.61	3.33	0.585

Data are reported as means (SD). *p*-values are reported for independent T-tests for means and chi-square values for nominal data. Bold highlight indicates significant *p*-values. CGAS/GAS = Children or Adult Global Assessment Scale, combined into a single measure; SES = Hollingshead Two-Factor Index of Social Class; PPVT IQ = Peabody Picture Vocabulary Test, used as a proxy for intelligence.

**Table 2:** Overall Volumes of the Hippocampus and Amygdala.

Volume	High Risk (n = 81)	Low Risk (n = 67)	df	T	*p*-Value
Whole Brain (cm^3^)	1267.5 (157.5)	1298.7 (131.3)	146	1.29	0.19
Right Hippocampus (mm^3^)	3164 (364.0)	3218.2 (366.3)	146	0.90	0.37
Left Hippocampus (mm^3^)	3098.9 (381.6)	3172.3 (360.6)	146	1.19	0.23
Right Amygdala (mm^3^)	1506.4 (261.4)	1463 (238.4)	146	1.04	0.29
Left Amygdala (mm^3^)	1505.5 (253.9)	1497.5 (225.4)	146	0.20	0.84

All mean values (+/−SD) are corrected for age, a quadratic term for age (age^2^), sex, and whole brain volume.

**Table 3:** Adjusting for the Non-Independence of Data on Group Differences.

	Adjusted Beta (Risk Group)	*p*-Value	Adjusted Beta (Risk-by-Sex)	*p*-Value
Unadjusted for Familial Correlations	−0.59	0.006	−0.02	0.95
Corrected with GEE	−0.567	0.002	−0.006	0.98
Corrected with Mixed Model	−0.3	0.0057	0.003	0.94

The identified voxel was located on the dorsal aspect of the left hippocampal head. GEE = Generalized Estimating Equations; Mixed Model = linear mixed effects model. The findings indicate that familial relatedness did not substantially alter the main effect of risk or the interaction of risk-by-sex on surface distances.

**Table 4:** Psychotropic Medication Use at the Time of MRI Scanning.

Medication Class	High Risk (n = 77)	Low Risk (n = 65)
Antidepressant	17	5
Anticonvulsant	5	0
Stimulant	1	1
Benzodiazepines	4	1
Sleeping Pill	1	0
Neuroleptic	0	0
Any psychotropic medication	20	6

## Data Availability

The data presented in this study are available freely upon written request from the corresponding author. We will require written agreement that the data will be used for research purposes only, will be performed with appropriate IRB and privacy protections in place, and will not be shared with a third party. We will request written agreement that the data will not be placed in a public repository, because the data are of human MRI scans from which faces and other identifying information can potentially be extracted, thereby placing individual privacy at risk. Furthermore, the written agreement will allow us to maintain a record of access to the dataset.
